# Predicting invasion success in complex ecological networks

**DOI:** 10.1098/rstb.2008.0286

**Published:** 2009-06-27

**Authors:** Tamara N. Romanuk, Yun Zhou, Ulrich Brose, Eric L. Berlow, Richard J. Williams, Neo D. Martinez

**Affiliations:** 1 Department of Biology, Dalhousie University1355 Oxford Street, Halifax, Nova Scotia B3H 3J5, Canada; 2 Pacific Ecoinformatics and Computational Ecology LabBerkeley, CA 94703, USA; 3 School of Public Policy, University of Maryland4113F Van Munching Hall, College Park, MD 20740, USA; 4 Department of Biology, Darmstadt University of TechnologySchnittspahnstrasse 10, 64287 Darmstadt, Germany; 5 Sierra Nevada Research Institute, University of California, MercedWawona Station, Yosemite National Park, CA 95389, USA; 6 Microsoft Research Ltd7 J. J. Thomson Avenue, Cambridge CB3 0FB, UK

**Keywords:** food web, invasibility, generality, nonlinear bioenergetic dynamic model, niche model, species invasions

## Abstract

A central and perhaps insurmountable challenge of invasion ecology is to predict which combinations of species and habitats most effectively promote and prevent biological invasions. Here, we integrate models of network structure and nonlinear population dynamics to search for potential generalities among trophic factors that may drive invasion success and failure. We simulate invasions where 100 different species attempt to invade 150 different food webs with 15–26 species and a wide range (0.06–0.32) of connectance. These simulations yield 11 438 invasion attempts by non-basal species, 47 per cent of which are successful. At the time of introduction, whether or not the invader is a generalist best predicts final invasion success; however, once the invader establishes itself, it is best distinguished from unsuccessful invaders by occupying a lower trophic position and being relatively invulnerable to predation. In general, variables that reflect the interaction between an invading species and its new community, such as generality and trophic position, best predict invasion success; however, for some trophic categories of invaders, fundamental species traits, such as having the centre of the feeding range low on the theoretical niche axis (for non-omnivorous and omnivorous herbivores), or the topology of the food web (for tertiary carnivores), best predict invasion success. Across all invasion scenarios, a discriminant analysis model predicted successful and failed invasions with 76.5 per cent accuracy for properties at the time of introduction or 100 per cent accuracy for properties at the time of establishment. More generally, our results suggest that tackling the challenge of predicting the properties of species and habitats that promote or inhibit invasions from food web perspective may aid ecologists in identifying rules that govern invasions in natural ecosystems.

## 1. Introduction

The Global Invasive Species Database (http://www.issg.org) suggests that there are no natural rules that govern the processes of invasion, which have any real predictive value (Bright 1998) because any ‘generalizations about invaders over too wide a taxonomic range, such as all species, or all insects, or all angiosperms, invariably (lead to) too many exceptions to be useful’ (Williamson 1996). They list propagule pressure, suitability of the habitat and previous success in other invasions (Williamson 1996) as the only predictors of invasion success, which are of general predictive value. Similarly, Jeschke & Strayer (2006) have suggested that the only consistent predictors of invasion success for mammals, birds and fishes are propagule pressure and human affiliation. Despite this well-founded pessimism, developing better models to predict when and where species will successfully invade is imperative (Enserink 1999).

Here, we describe a successful exploration for general rules that govern invasion success within models of ecological networks that integrate models of food web structure and nonlinear dynamics (Williams & Martinez 2004*b*; Brose *et al*. 2005, 2006). The biological foundation of these models is the fundamental requirement of metabolic energy for all life (Brown *et al*. 2004) and organisms' different feeding roles in consuming and providing that energy (Pascual & Dunne 2006). Based on this foundation, we develop a food web theory of species' invasions, which describes how each invader's trophic function is mediated by other species' trophic activities and the structural topology of the invaded food web. Our hope is that general rules governing invasion success in Nature will emerge from computational explorations of invasions in theory.

Our approach builds on an extensive literature on structural food web models (Cohen *et al*. 1990; Dunne 2006) from which we select the niche model (Williams & Martinez 2000), an empirically successful stochastic food web model that uses species richness (*S*) and directed connectance (*C*) as input parameters to construct food webs (Williams & Martinez 2000, 2008; Dunne *et al*. 2004; Stouffer *et al*. 2006). Our work also uses a nonlinear model (Brose *et al*. 2005; Martinez *et al*. 2006) that extends earlier work on bioenergetic dynamics (Yodzis & Innes 1992; McCann & Yodzis 1995; McCann & Hastings 1997; McCann *et al*. 1998) to *n* species and arbitrary functional responses (Williams & Martinez 2004*b*). The niche model successfully predicts the network structure of the largest and most complex food webs in the primary literature (Williams & Martinez 2000, 2008; Camacho *et al*. 2002; Dunne *et al*. 2004; Stouffer *et al*. 2007). The bioenergetic model of population dynamics component successfully simulates persistent and non-persistent stable, cyclic and chaotic dynamics (Williams & Martinez 2004*b*) that are often found in natural systems (Kendall *et al*. 1998) as well as in experimental microcosms (Fussmann *et al*. 2000; Shertzer *et al*. 2002). Invasions are simulated by integrating the structural niche model with the extended model of bioenergetic dynamics to create models of ecological networks with and without an invader. Prediction of invasion success within our simulations is evaluated using fundamental-niche properties of the invasive species that are independent of interactions with its biotic environment, realized-niche properties of the invader, which capture the trophic interactions between the invader and its biotic environment, and food web properties of the biotic environment independent of the invader. This approach allows us to explore many different structural and dynamic aspects of invasion success before and after invasion attempts.

## 2. Material and methods

**Figure 1:**
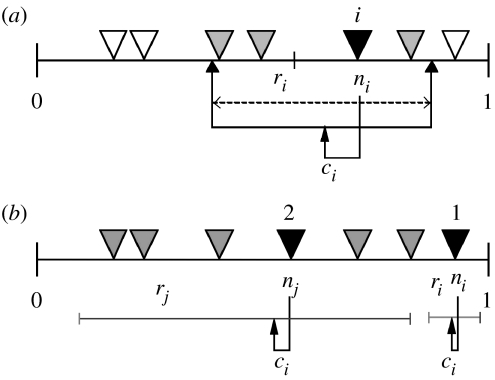
Diagram of the niche model and the invasion sequence. (*a*) *S* (trophic species richness) and *C* (connectance) are set at the desired values. Each of *S* species (here *S*=7, shown by inverted triangles) is assigned a ‘niche value’ (*n*_*i*_) drawn uniformly from the interval (0, 1). Species *i* consumes all species falling in a range (*r*_*i*_) that is placed by uniformly drawing the centre of the range (*c*_*i*_) from the interval (*r*_*i*_/2, *n*_*i*_). Thus, in this diagram, species *i* consumes four species (grey and black triangles) including itself. The size of *r*_*i*_ is assigned by using a beta function to randomly draw values from the interval (0, 1) whose expected value is 2*C* and then multiplying that value by *n*_*i*_ to obtain a web with *C* that matches the desired *C*. These rules stochastically assign each invader three fundamental niche values (*n*_*i*_, *r*_*i*_, *c*_*i*_). These values determine the invader's fundamental niche and, in concert with the fundamental niches of species in the invaded web, determine the realized niche of the invader. Thus, for example, an invader *i* with a specific *r*_*i*_ and *c*_*i*_ has higher generality in an invaded web when relatively many species' *n*_*j*_ fit within *i*'s feeding range than when invading a web with relatively few species' *n*_*j*_ fitting within *i*'s *r*_*i*_. (*b*) Example of attempted invasions by two different invaders into the same web. Invader 1 cannot invade because no species fall within its feeding range. Invader 2 can invade as it has prey (five grey triangles and itself) and therefore is allowed.

‘Fundamental-niche’ properties of invasive species, hereafter called ‘invaders’, are characterized by three stochastically generated parameters that dictate the location of an invader *i* on a theoretical niche axis (*n*_*i*_) as well as the centre (*c*_*i*_) and width (*r*_*i*_) of *i*'s feeding range (Williams & Martinez 2000; figure 1). The simulated native community is characterized by 17 ‘web’ properties including species richness (*S*), connectance (*C*=*L*/*S*^*2*^ where *L* is the number of feeding relationships among species, Martinez (1991)) and 15 other network properties that describe the topological and trophic structure of the webs. The relationship between the invader and its newly invaded community is characterized by a five ‘realized’-niche properties, including the generality (*Inv-Gen*) and vulnerability (*Inv-Vul*) of the invader (e.g. the invaders normalized prey (generality) and predator (vulnerability) counts), which are determined by the interaction between an invader's ‘fundamental’-niche properties and the rest of the species' niches in the food web.

Our invasion simulations were constructed in three steps. The first step specifies the structure of food webs and the fundamental niche of the invaders using the niche model (figure 1; Williams & Martinez 2000). The second step computes the dynamics of a network for 2000 time steps (*t*=2000) using the structure–dynamic integrated model (Yodzis & Innes 1992; Williams & Martinez 2004*b*; Brose *et al*. 2005, 2006) to generate a collection of dynamically persistent webs. The third step adds invaders to persistent webs at *t*=2000 (time of introduction) and computes the dynamics of both invaded and corresponding uninvaded (control) webs for another 2000 time steps until *t*=4000 (time of establishment).

### (a) The niche model

The niche model (Williams & Martinez 2000, 2008; Dunne *et al*. 2004) uses species richness, *S*, and the connection probability of any pair of species known as directed connectance, *C* (*C*=*L*/*S*^2^, Martinez 1991) as input parameters to predict the structure of feeding relationships within a community (figure 1). Each *i*th of *S* species is assigned a randomly drawn ‘niche value’ (*n*_*i*_) from the interval (1, 0). Each *i*th species is then constrained to consume all prey species within a range of beta-distributed values (*r*_*i*_) whose mean is *C* and whose randomly chosen centre (*c*_*i*_) is less than the consumer's niche value. The placement of the feeding range allows up to half a consumer's range to include species with higher niche values than the consumer, thus allowing looping and cannibalism. The consumer is constrained to feed only on those species that fall within its feeding range. While several modifications of the niche model have been described (Cattin *et al*. 2004; Stouffer *et al*. 2005; Allesina *et al*. 2008), we continue to use the original niche model (Williams & Martinez 2000) because it appears to provide the most accurate overall fit to the empirical structure of complex food webs in terms of both the central tendencies and the variability of food web structure (Dunne *et al*. 2004; Fox 2006; Martinez & Cushing 2006; Stouffer *et al*. 2006, 2007; Williams & Martinez 2008). We generate invaders by parametrizing the niche model with *C*=0.15 and *S*=30 and then generating 100 species with this model to define each invader's corresponding *n*_*i*_, *r*_*i*_ and *c*_*i*_ without ever constructing the web that would result from these parameters.

### (b) Bioenergetic model of nonlinear food web dynamics

The dynamic model closely follows previous work (Yodzis & Innes 1992; McCann & Yodzis 1995; McCann & Hastings 1997; McCann *et al*. 1998) but is generalized to *n* species and arbitrary functional responses (Williams & Martinez 2004*b*; Brose *et al*. 2006; Martinez *et al*. 2006). Extending the earlier notation (Yodzis & Innes 1992) to *n*-species systems, the variation of *B*_*i*_, the biomass of species *i*, over time *t*, is given by

(2.1) $$\frac{\text{d}B_{i}(t)}{\text{d}t}=G_{i}(B)-x_{i}B_{i}(t)+\sum_{j}^{n}\left(x_{i}y_{ij}F_{ij}(B)B_{i}(t)-x_{j}y_{ji}F_{ji}(B)B_{j}(t)/e_{ji}\right).$$

The first term on the right-hand side *G*_*i*_(*B*)=*r*_*i*_*B*_*i*_(*t*) (1−*B*_*i*_(*t*)/*K*_*i*_) is the net primary production rate of a basal species *i*. *r*_*i*_ is the intrinsic growth rate that is non-zero only for basal species and *K*_*i*_ is the carrying capacity; the second term is metabolic loss where *x*_*i*_ is the mass-specific metabolic rate; the third and fourth terms are gains from resources and losses to consumers, respectively, where *y*_*ij*_ is the maximum rate at which species *i* assimilates species *j* per unit metabolic rate of species *i*; (1−*β*_*ij*_). The non-dimensional functional response that may depend on resource and consumer species' biomasses, *F*_*ij*_(*B*), gives the realized fraction of the maximum ingestion rate of predator species *i* consuming prey species *j*; *e*_*ij*_ is the conversion efficiency with which the biomass of species *j* lost due to consumption by species *i* is converted into the biomass of species *i*. Dividing the last term by *e*_*ij*_ converts the biomass assimilated by consumer *j* into biomass lost by resource *i*. The parameters in these equations have been estimated from empirical measurements (Yodzis & Innes 1992; Brose *et al*. 2005, 2006). We simplified the dynamic model by assigning the following fixed parameter values: *K*_*i*_=1; *r*_*i*_=1; *x*_*i*_=0.5; *y*_*ij*_=6; *e*_*ij*_=1; and *B*_*0ij*_=0.5. Previous analyses using this integrated structural–dynamic approach (Martinez *et al*. 2006) have shown that simulations that draw these parameters from normal distributions with specified means and standard deviations (*e*_*ij*_>1 not allowed) gave similar results to fixed parameter simulations.

While a wide variety of functional responses have been proposed in the literature (Holling 1959*a*; Hassell & Varley 1969; Beddington 1975; DeAngelis *et al*. 1975; Skalski & Gilliam 2001), our model uses a modified ‘type II.2’ functional response that is close to a type II response while being intermediate between type II and III functional responses (Holling 1959*b*) and providing much of the stability of type III response (Williams & Martinez 2004*b*). This response models consumption of resource *j* by consumer *i* as

(2.2) $$F_{ij}(B)=\frac{B_{j}^{1+q}}{\sum_{k}B_{k}^{1+q}+B_{0}^{1+q}},$$

where *B*_0_ is the half saturation density and *q*=0.2 (equation (2.2) is a type III response when *q*=1). A consumer consumes all available resources at a rate equal to its maximum consumption rate times the functional response. The amount that each resource loses to that consumer is equal to the resource's density divided by the sum of all the densities of the consumer's resources times the consumer's rate of consumption. This allows a generalist to consume at its maximal rate even if only one of its prey species has high biomass (Williams 2008). Such biologically reasonable consumption rates are much higher than usual. Instead, most commonly used functional responses limit generalists to consuming any one resource species to no more than their total maximum consumption rate divided by the number of the consumer's prey species no matter how abundant that one prey species is (e.g. Martinez *et al*. 2006; Rooney *et al*. 2006, see Williams 2008).

### (c) Generating persistent webs

We constructed three sets of 250 niche model webs with *S*=30 and *C*=0.05, 0.15 or 0.30 (total 750 webs) as input parameters. Webs with biologically implausible energy flow patterns, such as loops with no external energy source (e.g. cannibals with no other food source), were excluded from further consideration. This yielded 171, 162 and 147 webs, respectively, for each of the three categories of *C* (0.05, 0.15 and 0.30). We then assigned each species a uniformly random biomass between 0.5 and 1 at *t*=0 and simulated the dynamics of these webs until *t*=2000. Species were eliminated from the web if their abundance decreased below our extinction threshold of 10^−10^. Mean species richness (s.d.) of the webs at *t*=2000 is 22 (3.66), 15 (4.44) and 10 (4.70) for each group of webs among *C*=0.05, 0.15 and 0.30 webs, respectively. Fifty webs with *S*≥15 were randomly selected and labelled (*w*) from 1 to 50 for each of the three *C* categories (0.05, 0.15 and 0.30) resulting in a total of 150 persistent webs that were used for further simulations.

### (d) Invasion simulations

Each invasion was simulated by adding one invader with a uniformly random initial biomass (*Inv-Bio*) between 0.5 and 1 to a persistent food web at *t*=2000. Invaded webs were discarded if the invader was trophically unconnected to the invaded web or if the invasion caused biologically implausible energy flows such as the loops discussed above. Acceptable invaded webs and the same webs lacking invaders continued to be dynamically simulated until *t*=4000, employing the same extinction threshold applied during *t*<2000. The webs without invaders will be used in future work exploring the effects of invasions on webs and will not be considered further here. We repeated this procedure for each of 150 persistent webs with the 100 model invaders yielding a total of 15 000 invasion scenarios. This set of 15 000 invasion scenarios yielded 11 483 invasions after eliminating those with biologically implausible food web structures (196 cases) and those where the invader was a basal species (3231 cases). Invasions by basal species were eliminated because the constant growth term of basal species' within our model makes it almost impossible for basal species to go extinct and usefully inform our study of differential invasion success. All simulations are programmed in Java v. 1.4.1 and run using fifty 64 bit processors operating within a large cluster running Linux 2.4.20. These methods generate a wide range of food webs being invaded by a wide range of invaders that allows an unusually thorough exploration of how the properties of the webs and invaders interact to determine invasion success.

### (e) Food web and invader properties

We use 17 properties to describe food web structure. One property is simply the number of species within the food web (*S*). Two other properties are standard measures of food web trophic interaction richness (Martinez 1991, 1992): links per species (*L*/*S*) also referred to as link density; and directed connectance (*C*=*L*/*S*^*2*^) that equals the proportion of all possible trophic links that are actually realized. Five more properties indicate the fraction of the following types of species in a food web: top (%*T*, species that have resource species but lack any consumer species, Briand & Cohen (1984)); intermediate (%*I*, species that have both resource and consumer species, Briand & Cohen (1984)); basal species (%*B*, species that have consumer species but lack resources species, e.g. plants, Briand & Cohen 1984); cannibals (*%C*, species that eat themselves, Williams & Martinez (2000)); and omnivores (%*Omn*, species that eat species at different trophic levels (*TL*s), Williams & Martinez (2000)). Four more properties indicate the fraction of links between the following types of species (Cohen & Briand 1984): top–basal (*%T–B*); top–intermediate (*%T–I*); intermediate–intermediate (*%I–I*); and intermediate–basal (*%I–B*). Mean *TL* was calculated in two ways (Williams & Martinez 2004*a*). One is the mean of the shortest chain length (*SCL*) between each species and basal species (*MSCL*). The other is the mean of each species' prey-averaged *TL* (*MTL*) that is the mean of each species' resource species plus 1. Two additional properties are the standard deviation of mean generality (*GenSD*) and vulnerability (*VulSD*) among species which quantify the variabilities of species' normalized predator and prey counts, respectively (Schoener 1989; Williams & Martinez 2000). Trophic similarity of a pair of species is the number of predators and prey shared in common divided by the pair's total number of predators and prey (Martinez 1991, 1993*a*,*b*; Solow & Beet 1998; Yodzis & Winemiller 1999). The average of all species' similarity indices is the property called mean similarity (*MSim*). The final web property is a ‘small-world’ (Watts & Strogatz 1998) property called the clustering coefficient (*CC*), which is the mean fraction of species pairs connected to the same species and each other (Camacho *et al*. 2002; Dunne *et al*. 2002; Montoya & Solé 2002; Williams *et al*. 2002*a*,*b*).

Nine properties of the invader were evaluated. Three properties describe the fixed properties (*n*_*i*_, *r*_*i*_ and *c*_*i*_; figure 1) that characterize the invader's fundamental niche and five properties describe the realized niche of the invader within the invaded food web. The fundamental and realized niches of the invader differ because the invader's realized niche depends on the niches of other species' in the invaded web whereas the fundamental niche does not. The invader's realized-niche properties of generality (*Inv-Gen*), vulnerability (*Inv-Vul*), and omnivory (*Inv-Omn*) are defined as, respectively, the invaders normalized prey (generality) and predator (vulnerability) counts (Williams & Martinez 2000), and the standard deviation of the prey-averaged *TL* of the invader's prey (Williams & Martinez 2004*a*). Generality and vulnerability are normalized, respectively, by dividing the number of prey and predator species by the number of the species in the food web (*S*). Prey-averaged *TL* (*Inv-TL*) equals 1 plus the mean *TL* of all of the invader's resource species (Williams & Martinez 2004*a*). Short-chain *TL* (*Inv-SCL*) equals 1 plus the SCL from the consumer taxon to a basal taxon (Williams & Martinez 2004*a*).

The final invader property was the initial biomass of the invader (*Inv-Bio*), which was randomly assigned from a uniform distribution between 0.5 and 1. Initial invader biomass was, on average, 15 times higher than the equilibrium biomass of single species in the webs at *t*=2000, but was equivalent to the average initial biomass of each species in the webs (0.75) at *t*=0. Owing to the restricted range of values of invader biomass chosen, we expected minimal effects of invader biomass on invasion success.

Properties of the webs and invaders (when present) were calculated at *t*=0 (initial conditions), *t*=2000 (introduction) and *t*=4000 (establishment; electronic supplementary material). All 3321 invasions by basal species (*SCL*=0) were successful and, as mentioned above, removed from our results prior to the analyses described below. Unsuccessful invaders persisted an average of 199 time steps (s.d.=195) ranging from *t*=2030 to 3974.

### (f) Statistical analysis

We used discriminant analysis (DA) to statistically model invasion success. DA models responses of a categorical dependent variable, in this case invasion success or failure, in terms of a series of other properties that might influence such success. We define success and failure as the persistence or not (failure) of the invader maintaining more than 10^−10^ biomass for the 2000 time steps between *t*=2000 and *t*=4000. DA produces a set of coefficients that define the single linear combination of properties (the discriminant function), which best differentiates the successful from unsuccessful invasions. We used forward stepwise selection to choose the property that best discriminates between the successful and unsuccessful invasions at each step of the DA. This is akin to linearly regressing invasion success against each property and choosing the property whose regression explains the most variation in success. The residuals from that chosen regression are then evaluated in the next step to similarly choose the property that explains most of the variation of the residuals (i.e. yields the smallest Wilk's lambda) or, in other words, explains most of the variation unexplained by the previously chosen property. The procedure is stopped when none of the properties explain 5 per cent or more of the remaining variation.

DA was used to assess both which variables best predict invasion success and how accurate that prediction is given 27 potential properties of both invader and web observed at two different times: *t*=2000 (time of introduction) and *t*=4000 (time of establishment). The time of establishment model was based on the web and invader properties at *t*=4000 except for failed invaders in which case their properties are those at the time step that they dipped below the extinction threshold. Biomass of the invader was excluded as it would trivially explain all variation in success. The DA model was applied to several groups of invasions evaluated separately for introduction properties at *t*=2000 and establishment properties at *t*=4000. These groups include all 11 483 invasions across all *C*, groups for each *C* category separately (0.05. 0.15 and 0.3), for *t*=2000 and 4000, groups for four *SCL* and *TL* categories: non-omnivorous herbivores (*SCL*=1, *TL*=2); herbivorous omnivores (*SCL*=1, *TL*>2); secondary consumers (*SCL*=2); and tertiary consumers (*SCL*=3). The DA models for the *SCL* and *TL* categories excluded *Inv-TL* and *Inv-SCL*, which merely repeat the variables used to define the categories.

We tested the DA model's ability to predict invasion success by comparing discriminant functions from DAs of a randomly chosen 75 per cent of cases with discriminate functions from DAs of the remaining 25 per cent of cases. Discriminant functions estimated on one set of simulation results are expected to more poorly predict invasion success in a different set of results because the latter set plays no role in estimating the functions. We report results for DA models of the 75 per cent of cases unless specified otherwise as the cross-validation results that refer to the results from the 25 per cent of cases.

## 3. Results

### (a) Predicting invasion success from properties at time of introduction

**Figure 2:**
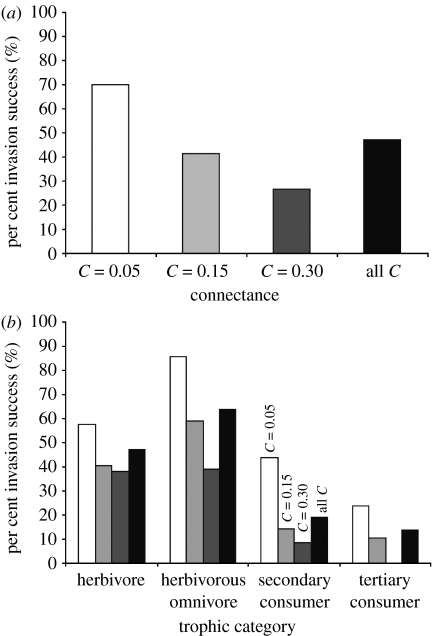
Invasion success. (*a*) Fraction of the total number of successful invasions across all *C* categories (black bars, all *C*) and in *C*=0.05 (white bars), 0.15 (light grey bars) and 0.30 (dark grey bars) webs. (*b*) Fraction of successful invasions according to trophic category across all *C* categories (black bars, all *C*) and in *C*=0.05 (white bars), 0.15 (light grey bars) and 0.30 (dark grey bars) webs. Trophic categories are defined according to the *SCL* and *TL* of the invader: herbivore (*SCL*=1, *TL*<2), herbivorous omnivore (*SCL*=1, *TL*>2), secondary consumer (*SCL*=2) and tertiary consumer (*SCL*=3). Fractions of successful and failed invasions are shown for *C*=0.05, 0.15 and 0.3.

Among all 11 483 analysed invasions, 47 per cent were successful (i.e. survive until *t*=4000; figure 2*a*). DA revealed that the properties at the time of introduction, which predict invasion success most accurately, are high generality (*Inv-Gen*), having the centre of the feeding range lower on the niche axis (*c*_*i*_), and a *SCL* leading to basal resources. Successful invaders also tended to invade webs with greater numbers of species (*S*) and webs with high variability of generality (*GenSD*) indicating that the webs are composed of both highly specialized and highly generalized species. A discriminant function using 15 out of the 27 properties retained in the stepwise DA discriminated between failed and successful invaders with 76.5 per cent accuracy (75% on cross-validation) and showed that invasion success was most accurately predicted by a relatively complex model that includes 8 out of the 18 web properties, 3 out of the 3 fundamental-niche properties of the invader and 4 out of the 5 realized-niche properties of the invader (table 1).

More insight into these general patterns comes from exploring in more detail how invasion success is affected by the highly variable properties of the invaded webs and the invader itself. Food web properties varied among the webs' initial values (*t*=0), their values at persistence (*t*=2000; Martinez *et al*. 2006) when the webs were invaded and their values at *t*=4000 when the invasion scenarios end (electronic supplementary material). Realized-niche properties of the invader also varied between their introduction at *t*=2000 and persisting or not until *t*=4000 (electronic supplementary material). We focus our results on the following questions: (i) which and how well properties of webs and invaders at the time of introduction (i.e. *t*=2000) predict invasion success, (ii) which are the differences between such predictions and those based on properties at the time of establishment (*t*=4000), and (iii) do our results systematically vary according to trophic category?

#### (i) DA model for invasion success within C categories

Invasion success is over twice as high in webs with low *C* compared with those with high *C* decreasing from 70 per cent in the *C*=0.05 webs to 42 per cent in the *C*=0.15 webs to 27 per cent in the *C*=0.30 webs (figure 2*a*). Invader generality (*Inv-Gen*) most accurately predicted invasion success in all three *C* categories. As *C* increased, DA retained web properties less often and the invaders' fundamental- as opposed to realized-niche properties better predicted invasion success (table 1). The discriminant function using 14 out of the 27 properties retained for *C*=0.05 webs discriminated between failed and successful invaders with 72.5 per cent accuracy (70% on cross-validation). As connectance increased, the DA retained fewer variables dropping sharply to eight properties predicting invasion success with 74 per cent accuracy (76.5% on cross-validation) in the *C*=0.15 webs, to only six properties predicting success with 68 per cent accuracy (65.5% on cross-validation) in the *C*=0.30 webs. This increase in model parsimony occurs concurrent with the increased importance of fundamental-niche properties (5, 7 and 29% of variability explained among *C*=0.05, 0.15 and 0.30 webs, respectively) and decreased importance of web properties (18, 14 and 3% among *C*=0.05, 0.15 and 0.30 webs, respectively). Most importantly, the fundamental-niche property, *c*_*i*_, becomes a more accurate predictor of invasion success than the realized-niche property, invader generality (*Inv-Gen*) in *C*=0.30 webs. Despite these differences, properties relating to high invader generality remain the most accurate predictors of invasion success within all *C* categories.

#### (ii) DA models for invaders within trophic-level categories

Omnivorous herbivores were the most successful trophic category of invader with invasion success occurring in 64 per cent of cases followed by herbivores (47%), secondary consumers (19%) and tertiary consumers (14%; figure 2*b*). Both successful non-omnivorous (herbivores that only eat basal species (*SCL*=1, *TL*=2)) and omnivorous herbivores (*SCL*=1, *TL*>2) tend to invade webs with more species (*S*), have a fundamental-niche constraining them to eat low on the niche dimension (*c*_*i*_) and a realized-niche that includes more resource species (higher *Inv-Gen*). Successful non-omnivorous herbivores differ slightly from omnivorous herbivores by tending to invade webs with fewer numbers of links per species (*L*/*S*) whereas successful omnivorous herbivores tend to have larger fundamental feeding ranges (*r*_*i*_). The DA retained 15 out of the 27 properties and predicted invasion success by non-omnivorous herbivores with 72 per cent accuracy (73.5% cross-validation; table 2) and, for omnivorous herbivores, retained 14 properties that predicted success with 72.5 per cent accuracy (75% cross-validation).

Unlike herbivores, invasion success of primary carnivores (*SCL*=2) is most accurately predicted by a single realized-niche property: high invader generality (*Inv-Gen*). However, the DA also retained 11 out of the 18 web properties and a fundamental-niche property, *c*_*i*_, as significant, albeit very minor, predictors of invasion success. The DA for primary carnivores discriminated between failed and successful invasions with 75 per cent accuracy (71.5 per cent cross-validation).

Unlike lower trophic categories for which success is most accurately predicted by realized and fundamental-niche properties, invasion success of secondary carnivores (*SCL*=3) is most accurately predicted by web properties. Specifically, successful secondary carnivores invaded webs with few links between other top species and intermediate species (*%T–I*), webs that had fewer top species (*%T*) and cannibals (*%C*), and webs that have species with similar numbers of consumers (low *VulSD*). The DA for secondary carnivores discriminated between failed and successful invasions with 89 per cent accuracy (95 per cent cross-validation).

#### (iii) Invader biomass

*Inv-Bio* at *t*=2000 was unrelated to invasion success. Among successful invasions, both mean biomass of the species in the web and final *Inv-Bio* was the highest in *C*=0.05. *Inv-Bio* of these successful invaders' at *t*=4000 averaged 0.01 in the *C*=0.30 webs, 0.021 in the *C*=0.15 webs and 0.062 in the *C*=0.05 webs (electronic supplementary material). Final mean invader biomass was lower than final mean biomass for each species in the web for all *C* categories.

### (b) Predicting invasion success from properties at establishment

When the establishment-web and establishment-niche properties of the invasion scenarios are used (*t*=4000) to model invasion success across all *C* categories, the DA model discriminates between successful and failed invasions with 100 per cent accuracy (cross-validation 100%). Once established, accurate prediction of invasion success is dominated by fundamental and realized-niche properties of the invaders (table 3). Specifically, low *c*_*i*_, *Inv-TL* and *Inv-Vul* are the properties that most accurately predict invasion success. However, the most accurate predictors of invasion success also differ within the *C* categories. In *C*=0.05 webs, successful invaders are most accurately predicted by having a low *TL* (*Inv-TL*). In *C*=0.15 and 0.30 webs, the most accurate predictor of invasion success is invulnerability to predation (*Inv-Vul*). While web properties remain accurate, albeit weak, predictors of invasion success at *t*=4000, niche properties of the invader accounted for the majority of the predictive accuracy of the models for both among all *C* categories and within all *C* categories.

## 4. Discussion

As indicated by the high and repeatable explanatory abilities of the DA models, invasion success was accurately predicted among our simulations despite highly variable food web structure and invader identity. This was especially true based on the properties at the end of the simulations (*t*=4000) compared with those based on the properties at the start of the invasions (*t*=2000). At *t*=4000, fundamental-niche properties of the invader, realized-niche properties of the invader and web properties discriminated between successful and unsuccessful invasions with 100 per cent accuracy. However, the corresponding *t*=2000 model was also highly accurate with 76.5 per cent of successful and failed invasions predicted accurately.

To successfully invade a habitat, a species must successfully complete three steps: introduction; establishment; and spread (Jeschke & Strayer 2005). Our network simulations focused on the first and second steps: properties at introduction and those at establishment. At *t*=2000, the fundamental-niche properties of the invader described by *n*_*i*_*, r*_*i*_ and *c*_*i*_ interact with the niche properties of all other species in the web to create a set of realized-niche properties that describe how the invader initially interacts within the web. Final conditions at *t*=4000 represent the combined effects of the invader's fixed fundamental-niche properties, direct interactions with the invader's predators and prey, and indirect interactions with other species and the web as a whole.

The models for *t*=2000 and 4000 capture complementary information about how invaders succeed in invading complex ecological networks. The most accurate predictors of invasion success at *t*=2000 are having high generality (*Inv-Gen*), having the centre of their feeding range (*c*_*i*_) low on the niche axis, and being herbivorous (*SCL*=1, *TL*≥2). However, while trophic category remains a highly accurate predictor of invasion success at *t*=4000, generality is no longer as accurate a predictor of invasion success as vulnerability to predation except in low connectance webs. These results suggest several potential rules about invasion success. Perhaps the most obvious example is that interactions between an invading species and its new community ultimately determine invasion success. More surprising is that, even at time of introduction, it may be possible to relatively accurately predict invasions success from a food web perspective.

Recent reviews of invasion ecology have suggested that models for species invasions must include traits of both the species and the environment, while recognizing that the interaction between an invasive species and other species in community is what ultimately determines invasion success (Lodge 1993; Shea & Chesson 2002; Ruesink 2005). However, there also appear to be fundamental properties associated with successful invaders that are not as dependent on the interaction of an invader with its new community. In our simulations, the most important fundamental property was the location of the centre of a species feeding range (*c*_*i*_) on the theoretical niche axis, which was an accurate predictor of invasion success, particularly in webs with higher connectance. Specifically, at *t*=2000 in *C*=0.30 webs, *c*_*i*_ contributes 59 per cent of the explained variance to the model and, at *t*=4000, *c*_*i*_ contributes 27 per cent of the explained variance to the model across all *C* categories. While *c*_*i*_ tends more towards intermediate values in webs with higher *C* in order to keep the larger *r*_*i*_ from extending beyond the niche dimension, it is unclear why eating low on the niche axis is a more accurate predictor of invasion than the corresponding realized-niche properties such as *SCL* or *Inv-TL*. Realized-niche properties such as low *TL* (*Inv-TL*) were initially expected to account for practically all variability because fundamental-niche properties appear to have no other function with our models other than to define realized niches. Further exploration needs to determine the source of this curious result. Not all properties of species' niches were observed in our models. Unobserved properties including ratios and other functions of observed properties may be discovered the correlate strongly with *c*_*i*_ and better elucidate *c*_*i*_'s role in invasion success.

Invasion success decreases sharply as connectance increases from 70 per cent in the *C*=0.05 webs to 42 per cent in the *C*=0.15 webs to 27 per cent in the *C*=0.30 webs. This suggests that less complex food webs have less crowded niche space with less competition for food resources, which facilitates invasion success. The higher biomass of species in *C*=0.05 webs further supports this interpretation making it an important and intuitively appealing hypothesis to explore further. However, empirical variability in *C* is typically confounded by differences in methodology among different investigators of different food webs (Martinez 1991, 1993*b*). Fortunately, consistent methodology (Havens 1992; Martinez 1993*a*; Srinivasan *et al*. 2007), use of trophic species (Martinez *et al*. 1999; Srinivasan *et al*. 2007) and moderate amounts of sampling effort (Martinez *et al*. 1999) may yield surprisingly robust measures of ecological complexity in terms of *C*. Applying such improved methodology to more and less invaded natural ecosystems could form an important test of whether ecological complexity in terms of trophic connectance strongly and consistently affects invasion success as suggested by our study.

Trophic position, and in particular the *SCL* of the invader, which tends to describe the dominant energy pathway of species (Williams & Martinez 2004*a*), accurately predicts invasion success in the introduction phase of our simulated invasions. However, as the invasion progresses, having a low *SCL* becomes less important than simply occupying a low trophic position. The distinction being that some species with high trophic positions may have a low *SCL* even though they feed mostly on high-trophic-position prey. The transition of importance from *SCL* to trophic position reflects the success of some of the primary carnivores that were successful in 19 per cent of invasion events and the secondary carnivores that were successful in 14 per cent of invasion events, which had high *SCL* but relatively low trophic positions.

These surprisingly intuitive and relatively simple results contradicted our expectation that invasion success and failure among our simulations would be as highly idiosyncratic similar to invasions in Nature (Williamson 1996). That is, each invasion outcome was thought to depend on fine nuances of each situation with invasion success based on the topology of the food web, the invaders fundamental and realized-niches, and fundamental and realized-niches of the other species in the web. Such idiosyncrasy could essentially preclude predictive simple regularities among invasions in our simulations and in Nature. However, instead of such idiosyncrasy, regularities identified by only a few predictive variables appeared among our simulations during both the introduction and the establishment stages of the invasions.

### (a) Simulation robustness and further questions

The generality of our results are limited by the initial densities of invaders being randomly assigned from the same distribution as the initial densities of the species in the webs (e.g. between 0.5 and 1 for all species). This choice of such high initial invader biomass probably prevented us from detecting the effects of low invader biomass. Another explanation is that our observed insensitivity of invasion success to invader biomass mirrors the independence of invasion success to invader density found by recent meta-analyses that have found propagule pressure, but not invader density, to be one of the few general predictors of invasion success (Lockwood *et al*. 2005; Jeschke & Strayer 2006). Another possibility is that the relatively high initial invader biomass in our simulations insulates invaders from initial vulnerability to predators and weakens the role of invader vulnerability among properties at *t*=2000 compared with its role at *t*=4000. Large invader biomass increases the chance that invaders overcome a well-known strong resistance to invasions of single species at low densities (Post & Pimm 1983; Drake 1990; Hewitt & Huxel 2002). For example, Hewitt & Huxel (2002) manipulated the inoculation density of invaders in model food webs and found that low densities of invaders result in almost complete invasion resistance. Previous studies likewise set inoculation densities at arbitrarily low values relative to Lotka–Volterra equilibrium densities that mimic colonization by rare species (Post & Pimm 1983; Drake 1990). The assumption that invasive species are introduced at low densities does not conform to all empirical invasion scenarios (Carlton & Geller 1993). For example, empirical invasions where high densities of invaders are involved include the movement of entire assemblages with oyster culture transfers, hull fouling and ballast water release (Carleton 1985, 1987; Carlton & Geller 1993; Ruiz *et al*. 2000). When Hewitt & Huxel (2002) relaxed their assumption of low inoculation densities, they found that 50–60% of their single-species invasions were successful; closely reflecting our value (47%) for the proportion of successful invasions. In future work, we expect that invader biomass will play a stronger role in determining invasion success if initial invader biomasses are distributed at and well below the equilibrium biomasses of the species in the webs.

Another aspect of our simulations affecting the generality of our results is the nature of the persistent niche model webs that are invaded. Our persistent webs are trophically shorter (low mean *TL*) and fatter (more basal and herbivore species) than niche webs not subjected to dynamics, which makes them more similar to food webs found in Nature (Williams & Martinez 2004*a*, 2008; Martinez *et al*. 2006; Thompson *et al*. 2007). However, the niche model tends to create food webs with less variability (Williams & Martinez 2000; Dunne *et al*. 2004) and slightly more intervality (Williams & Martinez 2000, 2008; Stouffer *et al*. 2006) than those that are found in Nature. In addition, due to computational considerations, we used a range of species richness that is lower than is found in empirical webs. Finally, our simulations ignore trophically important traits of plant nutrient consumption (Brose *et al*. 2005), variable body size (Brose *et al*. 2006; Otto *et al*. 2007) and non-trophic interactions such as facilitation. Such differences between our simulations and natural systems may limit the accuracy and applicability of our results. However, the many parallels between our results and many results of invasions found in Nature (see the electronic supplementary material for more examples) suggest that our simulations usefully inform invasion ecology.

## 5. Conclusion

Despite the common assertion that, other than propagule pressure and human affiliation (Williamson 1996; Jeschke & Strayer 2006), there are no strong taxa or biome independent determinants of invasion success, we identify potential generalizations describing successfully invasive species and more easily invaded communities by simulating species invasions within complex ecological networks. In general, an invader has a high probability of success of it is a generalist, a herbivore, an omnivore, a consumer with a feeding range that is low on a theoretical niche axis, and is relatively invulnerable to predation. Webs relatively easy to invade had low connectance, high mean biomass and a greater numbers of species. Integrating a complex-food web perspective into models of species invasion allows us to search a wide range of details that might affect invasibility and find potential systematic generalities among invasive species and habitats that promote successful invasions.

## Figures and Tables

**Table 1:** DA by *C* at *t*=2000 among all simulations combined (all) as well as within each *C* category separately for web properties and fundamental and realized-niche properties of the invader for predicting invasion success showing effect size (*F*), *p*-value (*p*) and the standardized function coefficients (coeff.). (Blank spaces indicate that the variable was not selected in forward stepwise DA. Positive coefficients are associated with traits that are associated with invasion failure while negative values are associated with invasion success. *F*, failed invasion; *S*, successful invasion.)

	all *C*			*C*=0.05			*C*=0.15			*C*=0.3		
				
	*F*	*p*-value	coeff.	*F*	*p*-value	coeff.	*F*	*p*-value	coeff.	*F*	*p*-value	coeff.
*web properties*												
*S*	90.127	<0.001	−0.023				33.009	<0.001	−0.018			
*C*	5.936	0.015	−0.672									
*L/S*	20.811	<0.001	0.065	18.658	<0.001	−0.119				4.395	0.036	−0.018
*%B*												
*%O*	29.727	<0.001	0.282	33.583	<0.001	0.531						
*%I*	46.763	<0.001	0.546									
*%C*										25.633	<0.001	−0.382
*%T*				5.683	0.017	0.381						
*%T–B*	44.377	<0.001	0.327	10.173	0.001	0.208						
*%T–I*												
*%I–I*	70.305	<0.001	−0.432									
*%I–B*							52.888	<0.001	−0.798			
*GenSD*	88.399	<0.001	0.281									
*VulSD*				4.724	0.030	0.173						
*MSCL*												
*MTL*							88.909	<0.001	−0.357			
*Msim*				84.989	<0.001	4.676						
*CC*							4.933	0.026	−0.163			
*fundamental-niche properties*												
*n*_*i*_	7.738	0.005	−0.067	11.323	<0.001	−0.138						
*r*_*i*_	76.668	<0.001	−0.449	5.847	0.016	−0.237	15.318	<0.001	−0.363	6.505	0.011	−0.227
*c*_*i*_	180.891	<0.001	0.392	32.621	<0.001	0.279	116.256	<0.001	0.336	294.019	<0.001	0.569
*realized-niche properties*												
*Inv-Gen*	557.810	<0.001	−0.127	188.430	<0.001	−0.099	279.224	<0.001	−0.245	146.874	<0.001	−0.294
*Inv-Vul*				5.945	0.015	0.028						
*Inv-TL*	9.397	0.002	−0.032	10.272	0.001	−0.078						
*Inv-SCL*	123.971	0.000	0.176	60.903	<0.001	0.235	137.198	<0.001	0.198	20.257	<0.001	0.074
*Inv-OMN*	30.258	<0.001	−0.128	10.229	0.001	−0.135						
*Inv-Bio*												

eigenvalue	0.548			0.408			0.452		0.310			
canonical R	0.595			0.538			0.558		0.487			
Wilk's lambda	0.646			0.710			0.689		0.763			
*P*	<0.001			<0.001			<0.001		<0.001			

	*F*		*S*	*F*		*S*	*F*		*S*	*F*		*S*
per cent correct												
(analysis)	82		71	56		89	83		65	92		44
per cent correct (cross-validation)	82		72	50		90	76		77	90		41

**Table 2:** DA by *SCL* at *t*=2000 with web properties and fundamental and realized-niche properties of the invader for predicting invasion success showing effect size (*F*), *p*-value (*p*) and the standardized function coefficients (coeff.). (Blank spaces indicate that the variable was not selected in forward stepwise DA. Positive coefficients are associated with traits that are associated with invasion failure while negative values are associated with invasion success. *F*, failed invasion; *S*, successful invasion.)

	*SCL*=1 *TL*=2			*SCL*=1 *TL*<2			*SCL*=2			*SCL*=3		
				
	herbivores			herbivorous omnivores			secondary consumers			tertiary consumers		
				
	*F*	*p*-value	coeff.	*F*	*p*-value	coeff.	*F*	*p*-value	coeff.	*F*	*p*-value	coeff.
*web properties*												
*S*	130.129	<0.001	−0.032	168.026	<0.001	−0.026	15.751	<0.001	−0.010	3.986	0.049	0.022
*C*												
*L/S*	51.618	<0.001	0.116	166.535	<0.001	0.085						
*%B*							86.498	<0.001	−1.200			
*%O*	29.308	<0.001	0.387	6.941	0.008	0.208	11.662	0.001	0.263			
*%I*				16.231	<0.001	0.520						
*%C*							6.305	0.012	0.236	24.581	<0.001	−1.598
*%T*	12.345	<0.001	−0.528				21.876	<0.001	−0.852	38.087	<0.001	−5.140
*%T–B*	7.048	0.008	0.203				7.404	0.007	0.405			
*%T–I*										68.060	<0.001	7.545
*%I–I*	50.203	<0.001	−0.564	56.458	<0.001	−0.699	8.518	0.004	−0.379			
*%I–B*				14.546	<0.001	−0.287	17.137	<0.001	−0.644	19.660	<0.001	−1.560
*GenSD*	50.742	<0.001	0.358	50.551	<0.001	0.338						
*VulSD*				6.265	0.012	−0.120	20.548	<0.001	0.230	13.994	<0.001	1.793
*MSCL*	38.839	<0.001	0.419	18.043	<0.001	0.376						
*MTL*	19.211	<0.001	−0.162	42.904	<0.001	−0.263	91.117	<0.001	−0.344			
*Msim*	14.857	<0.001	−1.518									
*CC*							15.553	<0.001	−0.211			
*fundamental-niche properties*												
*n*_*i*_	8.208	0.004	−0.078	6.878	0.009	−0.080						
*r*_*i*_	116.813	<0.001	−0.671	126.561	<0.001	−0.747						
*c*_*i*_	374.795	<0.001	0.599	266.935	<0.001	0.555	50.356	<0.001	−0.211			
*realized-niche properties*												
*Inv-Gen*	291.168	<0.001	−0.108	157.167	<0.001	−0.088	846.182	<0.001	−0.298			
*Inv-Vul*							4.631	0.031	0.029			
*Inv-BIO*												
*Inv-OMN*	22.635	<0.001	−0.129									

eigenvalue	0.398			0.452			0.622		1.066			
canonical R	0.534			0.558			0.619		0.718			
Wilk's lambda	0.715			0.689			0.617		0.484			
*P*	<0.001			<0.001			<0.001		<0.001			

	*F*		*S*	*F*		*S*	*F*		*S*	*F*		*S*
per cent correct (analysis)	78		68	60		85	95		55	98		80
per cent correct (cross-validation)	78		67	65		85	96		47	92		100

**Table 3:** DA by *C* at *t*=4000 across all simulations combined (all) as well as within each *C* category separately for web properties and fundamental and realized-niche properties of the invader for predicting invasion success showing effect size (*F*), *p*-value (*p*) and the standardized function coefficients (coeff.). (Blank spaces indicate that the variable was not selected in forward stepwise DA. Positive coefficients are associated with traits that are associated with invasion failure while negative values are associated with invasion success. *F*, failed invasion; *S*, successful invasion.)

	all *C*			*C*=0.05			*C*=0.15			*C*=0.3		
				
	*F*	*p*-value	coeff.	*F*	*p*-value	coeff.	*F*	*p*-value	coeff.	*F*	*p*-value	coeff.
*Web properties*												
*S*	3.623	0.057	−0.001							15.488	<0.001	0.017
*C*	81.368	<0.001	−0.433							8.263	0.004	0.658
*L/S*				48.560	<0.001	−0.060				11.690	0.001	−0.046
*%B*	446.791	<0.001	0.573	45.095	<0.001	0.430	189.273	<0.001	0.687	188.004	<0.001	0.573
*%O*	29.164	<0.001	0.082				19.891	<0.001	0.120			
*%I*												
*%C*				21.063	<0.001	−0.279						
*%T*							50.220	<0.001	−0.408	106.963	<0.001	−1.114
*%T–B*										94.647	<0.001	0.712
*%T–I*							25.128	<0.001	0.194	41.896	<0.001	0.399
*%I–I*				4.640	0.031	0.057						
*%I–B*							15.490	<0.001	−0.134			
*GenSD*							7.832	0.005	0.044	9.788	0.002	−0.067
*VulSD*				10.317	0.001	0.060	6.899	0.009	−0.033			
*MSCL*	285.379	<0.001	0.354	10.833	0.001	−0.145	72.832	<0.001	0.334	172.947	<0.001	0.420
*MTL*	178.646	<0.001	0.092	162.422	<0.001	0.430	11.581	0.001	0.073	4.080	0.043	0.012
*Msim*	55.493	<0.001	0.449	48.353	<0.001	0.843						
*CC*	7.542	0.006	0.032							7.230	0.007	0.039
*fundamental-niche properties*												
*n*_*i*_	474.830	<0.001	−0.150	105.600	<0.001	−0.128	290.156	<0.001	−0.189	444.991	<0.001	−0.191
*r*_*i*_	23.574	<0.001	0.061	19.058	<0.001	0.122	26.552	<0.001	0.103	17.611	<0.001	0.063
*c*_*i*_	3069.823	<0.001	0.370	1249.502	<0.001	0.443	822.751	<0.001	0.304	1004.292	<0.001	0.271
*realized-niche properties*												
*Inv-Gen*	1477.759	<0.001	−0.048	493.735	<0.001	−0.044	442.934	<0.001	−0.080	800.336	<0.001	−0.188
*Inv-Vul*	1740.905	<0.001	−0.119	138.880	<0.001	−0.053	1153.384	<0.001	−0.167	2583.355	<0.001	−0.254
*Inv-TL*	2687.111	<0.001	−0.183	14 938.838	<0.001	−0.313	499.277	<0.001	−0.130	62.427	<0.001	−0.044
*Inv-SCL*	799.516	<0.001	−0.203				457.020	<0.001	−0.255	984.770	<0.001	−0.383
*Inv-OMN*	9.594	0.002	−0.029	133.757	<0.001	0.136	81.043	<0.001	−0.141	10.770	0.001	−0.050

eigenvalue	19.320			13.266			24.691			30.983		
canonical R	0.975			0.964			0.980			0.984		
Wilk's lambda	0.049			0.070			0.039			0.031		
*P*	<0.001			<0.001			<0.001			<0.001		

	*F*		*S*	*F*		*S*	*F*		*S*	*F*		*S*
per cent correct (analysis)	100		100	100		100	100		100	100		100
per cent correct (cross-validation)	100		100	100		100	100		100	100		100

## Supplementary Material

Filename electronic supplementary material A Tables

Filename electronic supplementary material B
