# Supplementary material for: Predicting invasion success in complex ecological networks
Source: Philos Trans R Soc Lond B Biol Sci. 2009 Jun 27;364(1524):1743–54. doi: 10.1098/rstb.2008.0286 (PMC2685429; doi:10.1098/rstb.2008.0286)
Supplement: Filename electronic supplementary material A — Tables [file rstb20080286s04.doc]

**ESM A: Supplementary Material: Tables**

Table 1. Mean, standard deviation (S.D.), and coefficient of variation (CV) in web properties with invader at *t*=2000 and *t*=4000.

|  | ***t* = 2000** | | | ***t*=4000** | | |
| --- | --- | --- | --- | --- | --- | --- |
|  | mean | S.D. | CV | mean | S.D. | CV |
| **C=0.05** | | | | | | |
| *S* | 22.043 | 3.627 | 0.165 | 21.469 | 4.350 | 0.203 |
| *C* | 0.071 | 0.013 | 0.183 | 0.071 | 0.014 | 0.197 |
| *L/S* | 1.559 | 0.331 | 0.212 | 1.512 | 0.362 | 0.239 |
| *%B* | 0.519 | 0.082 | 0.158 | 0.538 | 0.088 | 0.164 |
| *%O* | 0.236 | 0.105 | 0.445 | 0.225 | 0.111 | 0.493 |
| *%I* | 0.359 | 0.135 | 0.376 | 0.345 | 0.146 | 0.423 |
| *%C* | 0.086 | 0.05 | 0.581 | 0.085 | 0.052 | 0.612 |
| *%T* | 0.122 | 0.105 | 0.861 | 0.117 | 0.110 | 0.940 |
| *%T-B* | 0.171 | 0.221 | 1.292 | 0.194 | 0.251 | 1.294 |
| *%T-I* | 0.051 | 0.069 | 1.353 | 0.050 | 0.065 | 1.300 |
| *%I-I* | 0.33 | 0.164 | 0.497 | 0.316 | 0.178 | 0.563 |
| *%I-O* | 0.449 | 0.143 | 0.318 | 0.439 | 0.153 | 0.349 |
| *GenSD* | 1.395 | 0.237 | 0.170 | 1.435 | 0.245 | 0.171 |
| *VulSD* | 0.59 | 0.128 | 0.217 | 0.582 | 0.156 | 0.268 |
| *MSCL* | 0.533 | 0.134 | 0.251 | 0.499 | 0.113 | 0.226 |
| *MTL* | 1.717 | 0.201 | 0.117 | 1.674 | 0.198 | 0.118 |
| *Msim* | 0.056 | 0.016 | 0.286 | 0.060 | 0.022 | 0.367 |
| *CC* | 0.103 | 0.069 | 0.670 | 0.103 | 0.072 | 0.699 |
| ***C*=0.15** |  |  |  |  |  |  |
| *S* | 18.767 | 2.227 | 0.119 | 18.709 | 2.294 | 0.123 |
| *C* | 0.169 | 0.021 | 0.124 | 0.169 | 0.021 | 0.124 |
| *L/S* | 3.163 | 0.454 | 0.144 | 3.156 | 0.460 | 0.146 |
| *%B* | 0.367 | 0.08 | 0.218 | 0.369 | 0.082 | 0.222 |
| *%O* | 0.476 | 0.115 | 0.242 | 0.475 | 0.114 | 0.240 |
| *%I* | 0.587 | 0.101 | 0.172 | 0.587 | 0.100 | 0.170 |
| *%C* | 0.128 | 0.064 | 0.500 | 0.128 | 0.064 | 0.500 |
| *%T* | 0.046 | 0.05 | 1.087 | 0.044 | 0.046 | 1.045 |
| *%T-B* | 0.033 | 0.052 | 1.576 | 0.033 | 0.049 | 1.485 |
| *%T-I* | 0.061 | 0.07 | 1.148 | 0.059 | 0.068 | 1.153 |
| *%I-I* | 0.5 | 0.138 | 0.276 | 0.500 | 0.137 | 0.274 |
| *%I-O* | 0.406 | 0.101 | 0.249 | 0.407 | 0.101 | 0.248 |
| *GenSD* | 1.091 | 0.161 | 0.148 | 1.093 | 0.162 | 0.148 |
| *VulSD* | 0.561 | 0.151 | 0.269 | 0.564 | 0.153 | 0.271 |
| *MSCL* | 0.662 | 0.113 | 0.171 | 0.659 | 0.114 | 0.173 |
| *MTL* | 2.092 | 0.288 | 0.138 | 2.089 | 0.289 | 0.138 |
| *Msim* | 0.125 | 0.023 | 0.184 | 0.125 | 0.023 | 0.184 |
| *CC* | 0.394 | 0.104 | 0.264 | 0.394 | 0.103 | 0.261 |
| ***C*=0.3** |  |  |  |  |  |  |
| *S* | 17.271 | 2.03 | 0.118 | 17.270 | 2.030 | 0.118 |
| *C* | 0.317 | 0.036 | 0.114 | 0.317 | 0.036 | 0.114 |
| *L/S* | 5.46 | 0.82 | 0.150 | 5.460 | 0.820 | 0.150 |
| *%B* | 0.287 | 0.091 | 0.317 | 0.287 | 0.091 | 0.317 |
| *%O* | 0.604 | 0.119 | 0.197 | 0.604 | 0.119 | 0.197 |
| *%I* | 0.7 | 0.094 | 0.134 | 0.700 | 0.094 | 0.134 |
| *%C* | 0.285 | 0.096 | 0.337 | 0.285 | 0.096 | 0.337 |
| *%T* | 0.013 | 0.025 | 1.923 | 0.013 | 0.025 | 1.923 |
| *%T-B* | 0.006 | 0.016 | 2.667 | 0.006 | 0.016 | 2.667 |
| *%T-I* | 0.015 | 0.034 | 2.267 | 0.015 | 0.034 | 2.267 |
| *%I-I* | 0.619 | 0.134 | 0.216 | 0.619 | 0.134 | 0.216 |
| *%I-O* | 0.36 | 0.128 | 0.356 | 0.360 | 0.128 | 0.356 |
| *GenSD* | 0.898 | 0.131 | 0.146 | 0.898 | 0.131 | 0.146 |
| *VulSD* | 0.5 | 0.122 | 0.244 | 0.500 | 0.122 | 0.244 |
| *MSCL* | 0.74 | 0.135 | 0.182 | 0.740 | 0.135 | 0.182 |
| *MTL* | 2.438 | 0.451 | 0.185 | 2.438 | 0.451 | 0.185 |
| *Msim* | 0.218 | 0.022 | 0.101 | 0.218 | 0.022 | 0.101 |
| *CC* | 0.62 | 0.109 | 0.176 | 0.620 | 0.109 | 0.176 |

Table 2. Fundamental (*n_i_, r_i_, ci,*) and realized-niche properties of the successful and failed invaders at *t*=2000 for *C*=0.05, 0.15, and 0.30 webs. Also shown is the mean number of species lost during the invasion, mean number of prey per invader, and mean number of predators per invader.

| **Initial *C*** | ***C*=0.05** | | ***C*=0.15** | | ***C*=0.3** | |
| --- | --- | --- | --- | --- | --- | --- |
|  | **mean** | **S.D.** | **mean** | **S.D.** | **mean** | **S.D.** |
| **Fundamental-niche properties** |  |  |  |  |  |  |
| *n_i_* | 0.667 | 0.255 | 0.663 | 0.257 | 0.687 | 0.246 |
| *r_i_* | 0.164 | 0.107 | 0.166 | 0.108 | 0.171 | 0.109 |
| *c_i_* | 0.444 | 0.244 | 0.436 | 0.244 | 0.457 | 0.238 |
| **Realized-niche properties** |  |  |  |  |  |  |
| *Initial Inv-Gen* | 2.289 | 1.434 | 1.080 | 0.708 | 0.623 | 0.419 |
| *Initial Inv-Vul* | 0.792 | 0.574 | 0.703 | 0.550 | 0.747 | 0.496 |
| *Initial Inv-TL* | 2.665 | 0.613 | 3.091 | 0.821 | 3.286 | 0.939 |
| *Initial Inv-SCL* | 1.220 | 0.436 | 1.334 | 0.493 | 1.402 | 0.500 |
| *Initial Inv-Omn* | 0.297 | 0.241 | 0.283 | 0.232 | 0.239 | 0.232 |
| *Final Inv-Gen* | 1.925 | 1.814 | 0.617 | 0.913 | 0.228 | 0.450 |
| *Final Inv-Vul* | 0.529 | 0.578 | 0.300 | 0.516 | 0.219 | 0.461 |
| *Final Inv-TL* | 1.806 | 1.276 | 1.187 | 1.484 | 0.776 | 1.370 |
| *Final Inv-SCL* | 0.793 | 0.595 | 0.463 | 0.588 | 0.300 | 0.526 |
| *Final Inv-Omn* | 0.310 | 0.233 | 0.145 | 0.215 | 0.301 | 0.220 |
| *Initial Inv-Bio* | 0.749 | 0.142 | 0.754 | 0.144 | 0.752 | 0.145 |
| *Final Inv-Bio* | 0.058 | 0.085 | 0.014 | 0.039 | 0.004 | 0.017 |
|  |  |  |  |  |  |  |
| Mean # of species lost | 1.801 | 2.100 | 1.083 | 1.433 | 1.241 | 1.859 |
| Mean # prey | 3.995 | 2.839 | 3.588 | 2.477 | 3.483 | 2.457 |
| Mean # predators | 1.402 | 1.088 | 2.368 | 1.953 | 4.209 | 2.949 |

Table 3. Final mean biomass of each species (and S.D.) and mean final biomass of the invader (and S.D.) for all invasion scenarios for each *C* category at *t*=4000. Initial biomass (*t*=2000) of each species in the web was randomly assigned between 0.5 and 1. Initial biomass of each invader was randomly assigned between 0.5 and 1.

| ***C*** | **Mean biomass of  species in the web (*t*=4000)** | **Mean invader biomass** | **Number of**  **species** |
| --- | --- | --- | --- |
| 0.05 | 0.085 (0.02) | 0.062 (0.08) | 20.5 (4.4) |
| 0.15 | 0.053 (0.01) | 0.021 (0.04) | 17.8 (2.6) |
| 0.3 | 0.035 (0.007) | 0.015 (0.03) | 16.1 (2.6) |

Table 4. Correlation coefficients between fundamental and realized-niche properties of invaders at *t*=2000 and *t*=4000. The relationship between fundamental and realized niche properties helps evaluate the results of DA. For example (see Fig. 1), a large *r_i_* indicates a wide fundamental feeding range that is expected correlate with realized trophic generality. Also, *c_i_* indicates where *r_i_* is located on the niche axis with species at high trophic levels tending to be located higher on the axis. The lower *c_i_*, the lower *r_i_* is placed and the lower one expects the trophic level of species *i*. When the DA selects an explanatory variable, much variation of that could also be explained by highly correlated variables is removed. This reduces the likelihood of choosing the correlated variables in subsequent steps of the DA. However, if properties such as generality and trophic level are extremely important to invasion success, then correlated variables may be chosen at more than one step in the DA.. As expected, fundamental and realized niche properties were highly correlated (Table 5). For example, *r_i_* is highly correlated with *Inv-Gen* both at *t*=2000 (0.50) and at *t*=4000 (0.48). Also, *c_i_* is highly correlated with *Inv-TL* both at *t*=2000 (0.67) and at *t*=4000 (0.67) because species with higher *c_i_* tend to be at higher trophic levels. Such correlations changed only slightly if at all between *t*=2000 and *t*=4000.

|  |  | ***Inv-Gen*** | ***Inv-Vul*** | | ***Inv-TL*** | | ***Inv-SCL*** | ***Inv-Gen*** | ***Inv-Vul*** | ***Inv-TL*** | ***Inv-SCL*** |
| --- | --- | --- | --- | --- | --- | --- | --- | --- | --- | --- | --- |
|  |  | *t*=2000 | *t*=2000 | *t*=2000 | | *t*=2000 | | *t*=4000 | *t*=4000 | *t*=4000 | *t*=4000 |
| **Fundamental-niche properties** | | |  | |  | |  |  |  |  |  |
| *n_i_* |  | 0.26 | -0.5 | | 0.43 | | 0.27 | 0.24 | -0.45 | 0.38 | 0.27 |
| *r_i_* |  | 0.5 | -0.25 | | 0.13 | | -0.14 | 0.48 | -0.2 | 0.06 | -0.14 |
| *c_i_* |  | 0.19 | -0.23 | | 0.67 | | 0.48 | 0.14 | -0.19 | 0.67 | 0.48 |
| **Realized niche-properties** | | |  | |  | |  |  |  |  |  |
| *Inv-Gen* | *t*=2000 | 1 | -0.17 | | -0.04 | | -0.14 | 0.96 | -0.13 | -0.09 | -0.14 |
| *Inv-Vul* | *t*=2000 | -0.17 | 1 | | -0.17 | | -0.13 | -0.18 | 0.95 | -0.11 | -0.13 |
| *Inv-TL* | *t*=2000 | -0.04 | -0.17 | | 1 | | 0.61 | -0.08 | -0.14 | 0.97 | 0.61 |
| *Inv-SCL* | *t*=2000 | -0.14 | -0.13 | | 0.61 | | 1 | -0.15 | -0.12 | 0.63 | 1 |
| *Inv-Gen* | *t*=4000 | 0.96 | -0.18 | | -0.08 | | -0.15 | 1 | -0.14 | -0.11 | -0.15 |
| *Inv-Vul* | *t*=4000 | -0.13 | 0.95 | | -0.14 | | -0.12 | -0.14 | 1 | -0.08 | -0.12 |
| *Inv-TL* | *t*=4000 | -0.09 | -0.11 | | 0.97 | | 0.63 | -0.11 | -0.08 | 1 | 0.63 |
| *Inv-SCL* | *t*=4000 | -0.14 | -0.13 | | 0.61 | | 1 | -0.15 | -0.12 | 0.63 | 1 |

Table 5. ANOVA for effect of initial *C* on food web properties at *t*=2000.

|  | ***Adj. R^2^*** | ***SS*** | ***MS*** | ***F*** | ***p*** |
| --- | --- | --- | --- | --- | --- |
| *S* | 0.370 | 49107.307 | 24553.654 | 3371.481 | <0.001 |
| *C* | 0.939 | 103.559 | 51.780 | 88283.810 | <0.001 |
| *L/S* | 0.881 | 28780.844 | 14390.422 | 42504.959 | <0.001 |
| *%B* | 0.561 | 97.521 | 48.761 | 7351.158 | <0.001 |
| *%O* | 0.643 | 245.946 | 122.973 | 10353.991 | <0.001 |
| *%I* | 0.590 | 187.929 | 93.965 | 8272.531 | <0.001 |
| *%C* | 0.570 | 75.267 | 37.634 | 7604.262 | <0.001 |
| *%T* | 0.219 | 14.706 | 7.353 | 1606.572 | <0.001 |
| *%T-B* | 0.193 | 33.921 | 16.960 | 1370.710 | <0.001 |
| *%T-I* | 0.087 | 4.596 | 2.298 | 550.459 | <0.001 |
| *%I-I* | 0.382 | 147.173 | 73.586 | 3552.462 | <0.001 |
| *%I-B* | 0.143 | 25.771 | 12.885 | 961.990 | <0.001 |
| *GenSD* | 0.543 | 390.634 | 195.317 | 6824.833 | <0.001 |
| *VulSD* | 0.048 | 10.759 | 5.380 | 290.374 | <0.001 |
| *MSCL* | 0.383 | 135.465 | 67.732 | 3558.341 | <0.001 |
| *MTL* | 0.462 | 1078.903 | 539.452 | 4929.307 | <0.001 |
| *Msim* | 0.910 | 45.565 | 22.782 | 58216.380 | <0.001 |
| *CC* | 0.810 | 438.900 | 219.450 | 24411.324 | <0.001 |
