# Supplementary material for: Predicting invasion success in complex ecological networks
Source: Philos Trans R Soc Lond B Biol Sci. 2009 Jun 27;364(1524):1743–54. doi: 10.1098/rstb.2008.0286 (PMC2685429; doi:10.1098/rstb.2008.0286)
Supplement: Filename electronic supplementary material B [file rstb20080286s05.doc]

**ESM B**: Invasion success in the real world

Most predictive studies of invasions in natural systems have focused specifically on predicting invasion success within particular taxonomic groups such as fish (Moyle & Light 1996; Kolar & Lodge 2002) or plants (Mitchell & Power 2003) and have focused heavily on the usefulness of life history traits, propagule pressure, or traits of the invaded habitat (Shea & Chesson 2002, Mitchell & Power 2003, Lockwood *et al.* 2005, Jeschke & Strayer 2006). In contrast, we tackled invasion prediction from a taxonomically and functionally broad food-web perspective; a perspective that may help to identify traits of highly invasive species or highly invasible communities that could apply to invasion scenarios less restricted by taxonomic affiliation, functional role, or ecosystem type. Below, we explain and discuss our results by identifying natural analogues that corroborate our working hypothesis that our i*n silico* explorations illuminate invasion processes that occur in nature.

Properties describing the width and location of the feeding niche were by far the best predictors of invasion success. This echoes the positive correlation between diet breadth and invasion success found in birds, mammals, and fish (Jeschke & Strayer 2006) among others. For example, the apple snail (*Pomacea canaliculata*), a freshwater snail from South America that has invaded and established in many tropical and sub-tropical regions is a generalist herbivore, eating most co-occurring water plants. Other common invaders with high generality are the black rat (Rattus rattus), the Nile perch (Lates nilcticus), the brown treesnake (Boiga irregularis), the starfish *(Asterias amurensis),* and the green crab *(Carcinus maenas).* Of the 66 animal invaders listed as the “worst invaders” in the Global Invasive Species Database, 95% are described as either generalists or omnivores. For example, the green crab (*C. maenas*) is listed as eating an “enormous variety of prey items, including organisms from more then 100 families and 158 genera in 5 plant and protist and 14 animal groups”.

The success of the green crab, which has successfully invaded estuaries and coastal shorelines throughout North America, Australia, and South Africa, exemplifies a key aspect of the realized properties of the invader beyond trophic generality that contributes to invasion success: the shortest chain length (*SCL*) of the invader, which reflects the fact that most of a population’s energy typically flows to it through its shorter paths to basal species (Williams & Martinez 2004). An *SCL*=1, which represents non-omnivorous and omnivorous herbivores, appears to be a hallmark of successfully invasive fish, mammals, and birds in North America and Europe (Jeschke & Strayer 2006). Shortest chain length provides information different from trophic level only in the case of omnivory where a species feeds on more than one trophic level. In our simulations, omnivorous and non-omnivorous herbivores are the most successful invaders. For both trophic groups, successful invaders have a fundamental-niche characterized by feeding lower on the niche axis, high realized generality, and are more successful at invading webs with higher *S*. Successful omnivorous herbivore invaders differ from strict herbivore invaders by being more successful in webs with fewer numbers of links per species (*L/S*).

Herbivory and omnivory appear to be a common property associated with high invasibility. For example, Ruesink (2005) examined patterns in fish invasions using FishBase and found that weighted by the number of cases, the likelihood of establishment of fish with different diet categories was omnivores (82%) ≥ zooplanktivores (78%) = benthivores (66%) = herbivores (63%) ≥ nekton feeders (54%). Many of the most successful invaders have been herbivores including: the primarily planktivorous zebra mussel (*Dreissena polymorpha*), goats (Capra hircus), which feeds on plants that are avoided by cattle and sheep and easily become feral, and the nutria (*Myocastor coypus*), a large herbivorous rodent originating from South America. Successful herbivorous omnivores include the European starling (*Sturnus vulgaris*), which consumes seeds, insects, invertebrates, plants, and fruit and the walking catfish (*Clarias batrachus*), which consumes detritus and opportunistically forages on large aquatic insects, tadpoles, and fish. Of the 66 animal invaders listed as “worst invaders” in the Global Species Invasion Database, 77% have an *SCL*=1 reflecting a basal source as their shortest chain while 23% have a *SCL*=2 indicating that their shortest chain was to herbivores. These values closely correspond to our results which showed that 60% of successful invasions had an *SCL*=1 and 19% had an *SCL*=2. This surprisingly close quantitative correspondence can be explained by the combination of similarity between the properties of both empirical and niche model webs (Williams & Martinez 2000; Dunne *et al.* 2004; Williams *et al.* 2002) and the types of species created in niche model webs and the types of species found in nature. While this latter correspondence between observed and theoretical species as opposed to between observed and theoretical webs is intuitive in the sense that it would be difficult to have the former without the latter, the species-level correspondence appears previously unrecognized.

One of the most interesting findings was that the specific mixtures of fundamental-niche properties, realized-niche properties, and web properties that contributed to invasion success in our simulations differ markedly among low and upper-trophic-position invaders. Herbivores, herbivorous omnivores, and secondary consumers showed strong regularities in the properties that contributed to invasion success with fundamental and realized-niche properties that captured information about trophic position and generality dominating the discriminating ability of the models. As the trophic position of the invader increased however, a different set of variables replaced the fundamental and realized-niche properties as the primary determinants of invasion success. For primary carnivores, invader generality contributed 70% of the explained variance to the model with the web properties of many basal species and low overall mean trophic level also accurately predicting successful invasion. In contrast, for secondary carnivores, fundamental or realized-niche properties were not retained in the model. For the secondary carnivores the only significant predictors of invasion success are web properties, particularly those suggesting reduced competition with other top species (i.e. *%* *T*, *%T-I*, *%C*, *VulSD*).

Intriguingly, the model for secondary carnivores was more parsimonious than the models for herbivores, herbivorous omnivores, or primary carnivores. Higher trophic levels invaders are almost guaranteed in our simulations to be successful if the web has a low overall mean trophic level, has a high diversity of herbivores and basal species, and lacks other top species. Successful invasions by secondary carnivores are often the result of intentional species introductions (Henneman & Memmott 2001) or occur on islands which often lack other top predators and typically have high diversity of intermediate consumers and basal species. For example, in Guam the introduction of the brown treesnake, a voracious generalist predator, has devastated the island food web resulting in multiple secondary extinctions (Fritts & Rodda 1998). Similarly, the black rat is now found on over 90% of the worlds island groups (Atkinson 1985). Once introduced, the generality of these species coupled with very few predator species enables extraordinarily high invasion success. Our results for invasion success for secondary carnivore invaders closely mirrors these and other invasion events in island food webs where the absence of other high-trophic-level species and high diversity of intermediate consumers lead to high rates of invasion success.

Other examples of successful invasions by secondary carnivores come from intentional species introductions. Many voracious fish species have been introduced into freshwater habitats as food for human consumption. Successfully introduced fish species such as the largemouth Bass (*Micropterus salmoides*) and the Nile perch, (L. nilcticus), which was introduced to Lake Victoria in 1954 for fisheries purposes, are highly piscivorous and cannibalistic but also prey on herbivores. These invasion events were also characterized by native food-webs which lacked other large-bodied piscivores. Another example of a successful carnivore invader is the cane toad (Bufo marinus) which eats nearly every terrestrial animal it comes across (Hinkley 1962) and as well as producing toxic secretions from its parotoid glands when it is provoked or localized pressure is applied to it, thus making it invulnerable to predators.

This latter property of the cane toad, invulnerability to predation, was also an accurate predictor of invasion success, especially in webs with intermediate and high connectance. Invader vulnerability is difficult to quantify in comparative empirical studies of consumers and as such has not been used in any statistical meta-analyses that attempt to predict invasion success (e.g. Kolar & Lodge 2001, Ruesink 2005, Jeschke & Strayer 2005). Despite this, anecdotal evidence for the importance of invulnerability is strong, particularly on islands that lack other large predators. For example, the brown tree snake, which has no natural predators on Guam, has attained densities of 2000 snakes/km^2^ (Fritts & Rodda 1998). The absence of natural enemies such as predators, parasites, and pathogens is often cited as a key determinant of invasion success (Mitchell & Power 2003, Torchin *et al.* 2003) and forms the basis for the use of biological control as a method to eradicate invasive species (Shea & Chesson 2002). Based on the ubiquity of biological control as a primary management strategy and the often cited importance of the enemy-release hypothesis (Shea & Chesson 2002), we expected invader vulnerability to be an important determinant of invasion success.

While invader vulnerability was the most accurate predictor of invasion success in at *t*=4000 in webs with *C*=0.15 and 0.30, invader vulnerability was a less accurate predictor in webs with *C*=0.05. The weaker accuracy of invader vulnerability in low connectance webs likely occurred because in *C*=0.05 webs an invader is, on average, only consumed by one predator. This makes low vulnerability more the norm rather than the exception. In contrast, in high connectance webs an invader is consumed, on average, by four predators. Our results suggest that low invader vulnerability may be a more accurate predictor of long-term establishment especially when it gives invaders more competitive advantage against native species such as those in more connected webs that are typically more vulnerable.

The properties of the web being invaded also contributed significantly to the models for invasion success. Web properties at *t*=2000 were more important than they were at *t*=4000. Successful invasions occurred more often in webs with greater numbers of species and in webs with lower connectance. Explorations of the roles of species richness in modulating invasion success (Elton 1958; Levine & D’Antonio 1999; Stachowicz & Tilman 2005) have suggested that whether species richness inhibits or facilitates invasions depends partially on the scale of observation (Levine 2000; Shea & Chesson 2002). At local scales, competition between species for resources appears to strongly suppress the establishment of invasive species (Stachowicz *et al.* 2002) which mirrors our connectance-related findings. At regional scales, species richness of invasive species is often correlated with native species richness because habitat conditions that nurture native species also nurture non-native species (Levine 2000; Shea & Chesson 2002). Within our limited range of species richness (*S*=15 to 26 species), species richness explains 6% of the variability in invasion success across all *C* categories in the models for both *t*=2000 and *t*=4000. This leads us to suggest that there are particular configurations of species, rather than simple numbers of species, within ecological networks that facilitates species invasions somewhat independent of the physical environment.

Literature Cited

Atkinson, I. A. E. 1985 The spread of commensal species of *Rattus* to oceanic islands and their effect on island avifaunas. In Conservation of island birds (ed. P. J. Moors). pp. 35-81 Cambridge: International Council for Bird Preservation (ICBP) Technical Publication No. 3.

Dunne, J. A., Williams, R. J., & Martinez, N. D. 2004 Network structure and robustness of marine food webs. *Marine Ecology Progress Series*, 273:291-302.

Elton, C. S. 1958 *Ecology of Invasions by Animals and Plants*. London, UK: Chapman & Hall.

Fritts, T. H., & Rodda, G. H. 1998.  The role of introduced species in the degradation of island ecosystems: A case history of Guam. *Annual Reviews of Ecology and Systematics*, 29:113-140.

Henneman, M. L., & Memmott, J. 2001 Infiltration of a Hawaiian community by introduced biological control agents. *Science*, 293:1314-1316.

Hinkley, A. D. 1962 Diet of the giant toad, *Bufo marinus* (L.), in Fiji. Herpetologica, 18: 253-259.

Kolar, C. S., & Lodge, D. M. 2002 Ecological predictions and risk assessment for alien fishes in North America. *Science*, 298:1233-1236.

Levine, J. M. 2000 Species diversity and biological invasions: relating local process to community pattern. *Science*, 288:852-854.

Levine, J., & D'Antonio, C. M. 1999. Elton revisited: a review of evidence linking diversity and invasability. *Oikos*, 87:15-26. Jeschke, J. M., & Strayer, D. L. 2005 Invasion success of vertebrates in Europe and North America. *Proceedings of the National Academy of Sciences (USA)*, 102:7198-202.

Jeschke, J. M., & Strayer, D. L. 2006 Determinants of vertebrate invasion success in Europe and North America. *Global Change Biology*, 12:1608-1619.

**Lockwood, J. L., Cassey, P., & Blackburn, T. 2005** The role of propagule pressure in explaining species invasions. ***Trends in Ecology and Evolution*,** 20:223-228.

Mitchell, C. E., & Power, A. G. 2003. Release of invasive plants from fungal and viral pathogens. *Nature*, 421:625-626.

Moyle, P. B., & Williams, J. E. 1990 Biodiversity loss in the temperate zone: decline of the native fish fauna of California. *Conservation Biology*, 4:275-284.

Moyle, P. B., & Light, T. 1996 Biological invasions of fresh water: empirical rules and

assembly theory. *Biological Conservation*, 78:149-161.

Post, D. M., Conners, M. E., and Goldberg, D. S. 2000 Prey preference by a top predator and the stability of linked food chains. *Ecology*, 81:8-14.

Ruesink, J. L. 2005 Global analysis of factors affecting the outcome of freshwater fish introductions. *Conservation Biology*, 19:1883-1893.

Shea, K., & Chesson, P. 2002 Community ecology theory as a framework for biological invasions. *Trends in Ecology and Evolution*, 17:170-176.

Stachowicz, J. J., Fried, H., Whitlatch, R. B., and Osman, R.W. 2002. Biodiversity, invasion resistance and marine ecosystem function: reconciling pattern and process. *Ecology*, 83:2575-2590.

Stachowicz, J. J., and Tilman, D. 2005. Species invasions and the relationships between species diversity, community saturation, and ecosystem functioning. In *Species Invasions: Insights into Ecology, Evolution, and Biogeography* (eds. D. F. Sax, J. J. Stachowicz and S. D. Gaines). pp. 41-64. Massachusetts: Sinauer Associates Inc.

Torchin, M. E., Lafferty, K. D., Dobson, A.P., McKenzie, V.J. and Kuris, A.M. 2003 Introduced species and their missing parasites. *Nature,* 421: 628-630.

Williams, R. J., & Martinez, N. D. 2004 Limits to trophic levels and omnivory in complex food webs: theory and data. *The American Naturalist*, 163:458-468.

Williams, R. J., & Martinez, N. D. 2000 Simple rules yield complex food webs. *Nature*, 404:180-183.

Williams, R. J., Dunne, J. A., & Martinez, N. D. 2002 Food-web structure and network theory: the role of connectance and size. *Proceedings of the National Academy of Sciences (USA)*, 99:12917-12922.
